# Tracing Back the Source of an Outbreak of Salmonella Typhimurium; National Outbreak Linked to the Consumption of Raw and Undercooked Beef Products, the Netherlands, October to December 2015

**DOI:** 10.1371/currents.outbreaks.1c667d62b51eb9840f5f7eb617e56bc1

**Published:** 2018-08-16

**Authors:** Gudrun Freidl, Stefanie Schoss, Margreet te Wierik, Max Heck, Paulien Tolsma, Anouk Urbanus, Ife Slegers-Fitz-James, Ingrid Friesema

**Affiliations:** EPIET fellowNational Institution for Public Health and the Environment; Netherlands Food and Consumer Product Safety Authority (NVWA); Consultant Communicable Disease ControlNational Institute of Public Health and the Environment; RIVMNational Institute for Public Helath and the Evironment; Public Health Servíce 'Brabant-Zuidoost'; National Institution for Public Health and the Environment; Netherlands Food and Consumer Product Safety Authority (NVWA), Utrecht, the Netherlands; Centre for Infectious Disease Control, National Institute for Public Health and the Environment (RIVM), Bilthoven, the Netherlands

**Keywords:** beef, Outbreak, Salmonella, trace-back

## Abstract

Introduction. On 23 October 2015, six related cases with gastroenteritis called the Netherlands Food and Consumer Product Safety Authority. They suspected filet américain, a raw beef spread, to be the source of infection. Leftovers and stool samples tested positive for Salmonella Typhimurium. Multiple locus variable-number of tandem repeat analysis (MLVA) revealed a MLVA pattern (02-23-08-08-212), which had not been detected in the Netherlands before. Concomitantly, an increase of this MLVA type was observed in the national Salmonella surveillance, amounting to 46 cases between 26 October and 9 December.

Methods. To investigate whether filet américain or an alternative (related) source could  be linked to surveillance-reported cases, cases (n=38) were invited to complete a questionnaire and upstream source tracing to map the food supply chain was initiated.

Results. Rapid interdisciplinary action resulted in identification of a contaminated 46-ton batch of beef distributed via a Dutch deboning plant as the likely source of infection. In total, 24/29 respondents (83%) could be linked to the incriminated batch of beef products (predominantly filet américain and minced beef).

Discussion. Repeated identification of raw meat products as a source of infection emphasizes the importance of awareness of the risk of infection when handling or consuming these products. Improved measures and procedures on product labelling, pre-treatment or product testing should be considered.

## Introduction

Salmonellosis is the second most reported zoonosis in the EU after campylobacteriosis^1^. Approximately 85% of human salmonellosis cases result from consumption of contaminated food, such as undercooked eggs, raw meat products, or raw fruit and vegetables^1^^,^^2^.

In the Netherlands, salmonellosis is only notifiable in case of a cluster with two or more human cases probably linked to contaminated food or drinking water^3^. Annually, approximately 15-20 *Salmonella* outbreaks are detected in the Netherlands^4^. Since 1987, fifteen regional public health laboratories together form the Dutch laboratory surveillance network for gastroenteric pathogens, which covers approximately 64% of the Netherlands^3^^,^^5^. As part of the *Salmonella* surveillance, these laboratories sent their *Salmonella* isolates to the National Institute for Public Health and the Environment (Rijksinstituut voor Volksgezondheid en Milieu, RIVM), where the National *Salmonella* Centre performs serotyping on submitted *Salmonella* isolates of human, animal or environmental origin.

In 2003 and 2012, the two largest recent national *Salmonella* outbreaks (~540 and 1149 laboratory-confirmed human cases) could be linked to contaminated eggs (*S*. Enteritidis) – imported during a large avian influenza virus outbreak – and salmon (*S*. Thompson)^4^^,^^6^. In 2009, raw beef products were epidemiologically strongly associated with an outbreak of *S*. Typhimurium with a novel phage type (23 cases), but microbiological analysis of incriminated meat products could not confirm the association^7^.

On 9 November 2015, an outbreak of Salmonella Typhimurium with a unique multiple locus variable-number of tandem repeat analysis (MLVA) pattern (02-23-08-08-212) was identified.

An outbreak investigation was started with the following aims (i) to find out whether cases of the local cluster and cases identified through the national surveillance shared a common or related source of infection and (ii) to trace the origin of the *Salmonella* contamination to prevent further cases and new outbreaks.

## Methods

**Case definition**

As *Salmonella* Typhimurium with MLVA pattern 02-23-08-08-212 had not been previously detected in the Netherlands, a case was defined as a person with laboratory-confirmed diagnosis for the outbreak *Salmonella* type. The sixth case within the local cluster was not laboratory-confirmed, but included as probable case due to the strong epidemiologic link with the other cases in the cluster.

**Microbiological investigation**

Strains isolated from human samples and from food products were detected and isolated by medical microbiological laboratories and the Netherlands Food and Consumer Product Safety Authority (Nederlandse Voedsel- en Warenautoriteit, NVWA), respectively. The method used for the food samples is based on the NEN-EN-ISO 6579/AI, which is an amendment to ISO 6579 published in 2007. Additionally to this method, the first enrichments (BPW) were screened for the presence of *Salmonella* based on the presence of the invA gene, followed by transferring positive samples to the second enrichment (MSRV). Different from the method in the amendment, selective plating media BGA and MLCB, instead of XLD, were used for the isolation of *Salmonella*. This method, including the screenings PCR, has been validated for horizontal use in food matrices.

At the RIVM, strains were serotyped based on O- and H-group antigens according to the World Health Organization (WHO) Collaborating Centre for Reference and Research on *Salmonella* standards^8^. *S*. Typhimurium were also typed by means of MLVA, which can discriminate between *S*. Typhimurium strains^9^.

**DNA testing of meat**

A *Salmonella* source attribution model, as described earlier^10^, pointed towards pigs/pork as the most likely source of *S*. Typhimurium with this MLVA type. To investigate whether filet américain might have been contaminated with pork, we tested the leftovers for the presence of pig DNA using real-time PCR with the Biorad CFX 96 system.

**Epidemiological investigation**

The surveillance-reported cases were invited to complete a questionnaire, either by phone or by mail conducted by the regional Public Health Services. As the index cluster provided a strong lead towards the most likely source of infection, we used a questionnaire tailored to consumption of various meat products [beef (raw ingredient of filet américain) and pork (indicated by source attribution model)] and venues where meat products were purchased (such as butchers or supermarkets). The questionnaire comprised 12 pre-listed supermarket chains. All questions focused on the 7 days prior to the onset of symptoms. Other items of the questionnaire included sections on demography, clinical symptoms, date of onset and duration of gastrointestinal illness, hospitalization and travel history. Data from questionnaires was entered into Microsoft Access (Microsoft Office Professional Plus 2010, Microsoft Corporation, Washington, USA) and analyzed using descriptive epidemiology. The analyses were conducted in SAS for Windows (SAS Institute Inc., Cary, NC, USA, version 9.3) and Microsoft Excel.

**Ethical considerations**

Medical Research Ethics Committee review and approval were not required as these kind of investigations are part of the routine public health response to an outbreak of salmonellosis. Cases were not asked for explicit informed consent, but indicated their willingness in participation in the interview. A case register including personal identifiers was used during the outbreak, which was needed for outbreak response and action. This register was only accessible by the outbreak management team who also performed all analyses.

**Trace-back**

During outbreaks, the NVWA is responsible for food trace back investigations according to Directive 2003/99/EC^11^. Supply chains of possible sources are examined based on available evidence from microbiological tests and/or epidemiological investigations. As decided in the EU Regulation (EC) 178/2002 (General Food Law), it is required that food products are traceable in two directions: one step forward (e.g. to the customer) and one step backward (e.g. to the supplier) in the food chain^12^. Immediately after the typing results revealed *S*. Typhimurium isolated from filet américain consumed by the local cluster, the NVWA launched an investigation to trace-back the origin of the contamination and surveyed whether contaminated meat products were still on the market. The investigation aimed to map the food supply chain from retail/catering premises to processors/slaughterhouse, to assess production hygiene, ‘Hazard Analysis and Critical Control Points’ (HACCP) and traceability.

## Results

**Start of the outbreak**

On 23 October 2015 (see timeline in Figure 1), a citizen living in the Southern part of the Netherlands, called the NVWA to report that in total six persons fell ill with gastrointestinal illness after having consumed filet américain, a bread spread consisting of finely chopped raw beef mixed with a herb sauce. A stool sample from one case of the cluster tested positive for Salmonella spp. On 29 October, stool samples from four of the five remaing cases tested positive for S. Typhimurium. On the same day, the NVWA detected S. Typhimurium in the filet américain leftovers, informed the regional Public Health Service ‘Hart voor Brabant’ who subsequently reported this local cluster to the RIVM.

On 9 November, typing results showed that the Salmonella isolates from the local cluster and the food leftovers had a unique MLVA-pattern (02-23-08-08-212). Furthermore, 26 additional cases were identified in that week. To check whether other European countries experienced a concurrent identical outbreak or had had an outbreak that could be linked to this one, an urgent inquiry was placed on the Epidemic Intelligence Information System for Food- and Waterborne Diseases and Zoonoses (EPIS-FWD) operated by the European Centre for Disease Prevention and Control (ECDC). This yielded no clues for solving the outbreak.

**Timeline of the outbreak of Salmonella Typhimurium in the Netherlands, October to December 2015.**
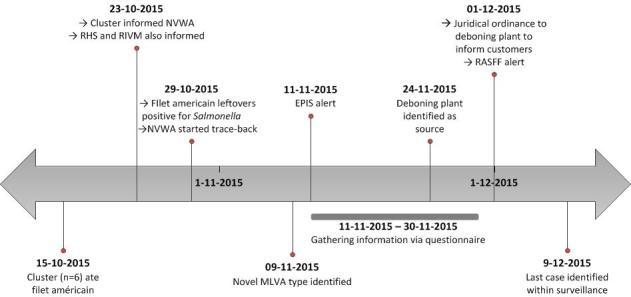
NVWA = Dutch Food Safety Authority; RHS = Regional Public Health Service; RIVM = National Institute for Public Health and the Environment

Between 26 October and 9 December 2015, 45 outbreak cases were laboratory-confirmed (including five from the index cluster of six cases). For 34 cases, the date of onset of illness was known (Figure 2); the majority (n=33; 97%) developed symptoms between 14 October and 21 October, whereas one case fell ill on 27 October. One case (4%) reported having been abroad for one day. The cases identified through the national surveillance (n=40) were distributed across the country. The majority of the laboratory-confirmed cases were female (n=26; 58%) and ages ranged from 2 to 79 years (median 21). Of these, 15 cases were 9 years of age or younger (33%), six cases were aged 10-17 years (13%), 16 were aged 18-49 years (36%) and eight cases were 50 years or older (18%).

**Epidemiological curve showing 34 cases with Salmonella Typhimurium infection by date of onset of illness in the Netherlands during October 2015.**
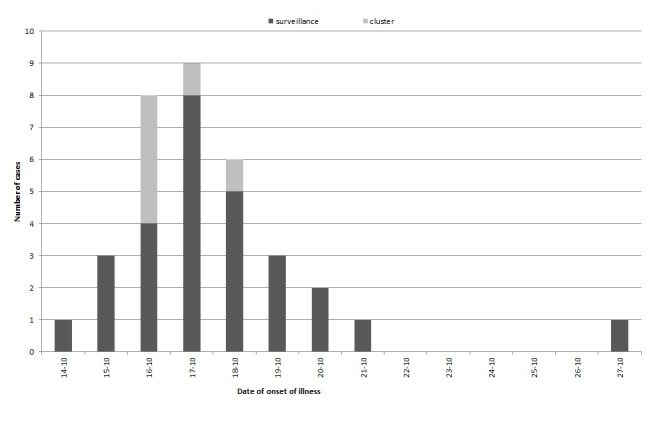
Six cases belong to the case cluster (5 thereof laboratory-confirmed), whereas the remaining depicted 28 cases – who completed a questionnaire – were identified through the national Salmonella surveillance system. For twelve other cases involved in this outbreak, date of onset of illness was not provided.

**Information from questionnaire**

The six cluster cases were not interviewed, as the only common source they have shared eating was filet américain and already a microbiological link with the filet américain was found for this cluster. All other cases typed until 23 November (n=38) were invited to fill in the questionnaire. Among those, 29 responded (response rate 76%). All 29 cases reported gastro-intestinal symptoms, eight (28%) were hospitalized. Diarrhoea lasted between 3 and 21 days (median: 9 days), based on information provided by 21 cases (72%).

Consumption of filet américain was most often reported (76%), followed by minced beef (55%). In total 26 cases (90%) ate one or both products in the week before illness. In a continuous survey ongoing since 2008^13^, around 20% of the Dutch general population reports eating filet américain in the past week, around 55% minced beef and 62% one or both products. Twenty cases (69%) reported to have purchased any meat products as part of their grocery shopping at a supermarket, two cases (7%) bought meat exclusively at the butcher (7%) and seven (24%) cases bought meat at both venues. Information on beef product, purchase date and purchase site was sent to the NVWA for the trace-back investigations.

**Trace-back investigations of food products**

The local cluster cases reported having purchased filet américain at supermarket chain D. Supermarket chain D produced filet américain locally by mixing prepared ground beef and a ready-made herb sauce. Inspection of the involved local production site did not reveal any breach of hygiene standards. Trace-back of the contaminated batch led to meat producer 1 (P1; Figure 3). P1 reported delivering 70% of its total meat supply to supermarket chain D. Raw meat linked to the contaminated filet américain was distributed to 300 branches of this chain. All raw material for filet américain at supermarket chain D originated from P1. These tracing efforts could link another 15 cases to supermarket chain D.

**Flow of trace-back operations of raw material contaminated with Salmonella Typhimurium, the Netherlands, October to December 2015:**
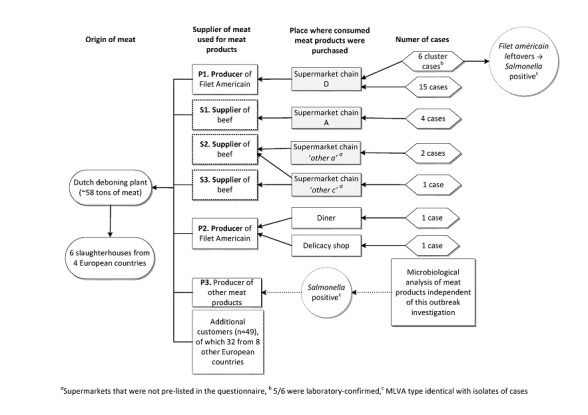

The raw material distributed by producer P1 was traced back to a Dutch deboning plant, which had processed ~58 tons of meat parts on 8 October 2015, originating from six slaughterhouses from four different European countries (Figure 3). In total, the deboning plant delivered ~46 tons of meat for human consumption to 55 meat-processing plants; of these, 32 were located in eight other EU Member States (~15 tons). Using information from forward and backward tracing, together with information from the questionnaires, we could establish links between the Dutch deboning plant and suppliers of three other supermarket chains, a diner and delicacy shop to which nine additional cases could be linked.

The NVWA – via juridical ordinance – commissioned the plant on 1 December 2015 to inform all customers that received parts of the contaminated batch. Dutch customers of the deboning plant were legally required to inform the NVWA how much of the contaminated meat they had processed and distributed. As the contaminated 46-ton batch of beef was mixed with meat from other parties at various customers, the contaminated batch amounted to 400 tons in total. Of those 400 tons, around 65 tons (16%) were classified as risk products defined as “ready to eat” products or products very likely to be consumed with a mild treatment, insufficient to eliminate or reduce the risk of infection by *Salmonella* to an acceptable level taking into account consumers’ consumption habits^14^. Most risk products had already been sold and/or the best before date had expired. About 1 ton was still available and withdrawn to prevent further cases.

**Microbiological testing of food products**

Besides the positive leftovers, samples of filet américain and separate samples of sauce and raw meat meant to be used for filet américain were taken at producer P1. All samples were from other batches than the incriminated batch, and tested negative for *Salmonella* spp. No samples were taken at the deboning plant as the particular batch was already distributed.

Although the source attribution model indicated that the outbreak MLVA type was more likely to be linked with pork/pigs (58%), than with beef/cattle (3%), no pig DNA was detected in the leftover filet américain.

## Discussion

Here we describe the investigation of a national outbreak of *S*. Typhimurium with MLVA type 02-23-08-08-212 linked to raw and undercooked beef products. Based on microbiological and epidemiological evidence, beef sold as filet américain and minced beef was the source of infection. Timely involvement of the different authorities and exchange of information regarding epidemiological, laboratory and trace-back results led to the identification of a 46-ton batch of beef. The batch was distributed via a Dutch deboning plant. The plant received its meat from slaughterhouses outside the Netherlands, and thus outside Dutch authority, and with *Salmonella* not being a food safety criteria on farm level, further trace back was not performed. As parts of this batch could be traced forward to eight other EU countries, an alert in the Rapid Alert System for Food and Feed (RASFF) was placed. Risk products that were still on the market were withdrawn to protect consumers.

‘Exposure to raw beef’ and ‘consumption of undercooked meat’ are known risk factors for *S*. Typhimurium infection^15^. In the Netherlands, filet américain constituted the likely vehicle of infection in three previous *S*. Typhimurium outbreaks^7^^,^^16^. Likewise, filet américain was also associated with outbreaks caused by Shiga toxin-producing Escherichia coli O157 in the past^17^^,^^18^^,^^19^. A previous Dutch outbreak caused by *S*. Typhimurium was linked to an outbreak in Denmark, which preceded the outbreak in the Netherlands^16^. In Denmark, cases were infected through consumption of carpaccio. Trace-back led to a contaminated batch of beef imported from Italy^20^. As the Dutch outbreak occurred after the Danish outbreak, this example shows that rapid source tracing and timely retraction of contaminated products from the market is vital to prevent further cases.

Although trace-back revealed a batch of 46 tons as contaminated, only 46 cases were reported. The identified batch is the production of one day in the deboning plant. It is therefore likely that the batch will not be contaminated completely or evenly due to a point source contamination of one or more carcasses. Secondly, the number of cases will be an underestimation as most people with gastroenteritis do not visit a physician or are not tested. It was estimated earlier that around 1 in 20 Salmonella patients will be laboratory-confirmed^25^^,^^26^ leading to approximately 900 outbreak cases in the general population.

In outbreak situations where the source of infection has a short shelf life, microbiological detection of the pathogen in the food source is often not possible as the product is no longer on the market when trace-back efforts commence. In the current outbreak, the alert by the local cluster allowed timely microbiological testing of leftovers of filet américain, which expedited the process of identifying the underlying source. Parts of the contaminated batch of filet américain were still on the market (expiry date products producer P1: 17 October 2015) and could be recalled. Besides early identification of an outbreak, collaboration between regional and national authorities, and public health and food authorities is imperative to find the source. Regular contact and exchanging of gathered information proved to be crucial. Trace-back efforts could link 24 of 29 interviewed national surveillance-reported cases to the contaminated batch of beef. Although leftovers cannot be regarded as formal evidence for the source of infection, the microbiological results provided a strong clue for the trace-back, which was later confirmed by the positive results of a survey done independent from the outbreak investigations. An animal welfare organization performed microbiological tests in mini hamburgers included in a package containing a selection of different types of meat for raclette grilling. These mini hamburgers tested positive for *S*. Typhimurium with identical MLVA type. Raw material used for this product was subsequently linked to the 46-ton batch distributed via the deboning plant (Figure 2).

The moment the national outbreak was detected, the local cluster could be linked to filet américain. Furthermore, the MLVA pattern had not been seen earlier in the Netherlands. Therefore, we refrained from conducting a classical case-control study and decided to tailor the questionnaire to meat products for two purposes. First, to confirm beef products as source of the outbreak and secondly, to gather information on type of beef products, purchase dates and purchase sites to feed the trace-back. This contributed to a timelier start of the trace-back investigation and a likely quicker withdrawal of incriminated meat. To check whether filet américain or other beef products indeed were eaten more often than expected, we used the national controlsurvey^13^. Although less accurate than a case-control study as the reports are not from the same week in time, it answered the need of confirmation. The increase in cases observed in the regular surveillance would have similarly resulted in an outbreak investigation, which would – most likely – have led to the same incriminated products and trace-back results. However, the process would have likely taken considerably longer, as first a trawling questionnaire would have needed to be administered followed by a case-control study.

Trace-back investigation should be undertaken wherever possible to identify the initial source of contamination as this can lead to better source attribution and provide further evidence for policy changes at primary production and processing to reduce the risk of *Salmonella* entering the food chain. However, it has to be acknowledged that trace-back operations remain cumbersome and time-consuming. During the trace-back operation, the complexity of the food supply chain and diverse stakeholders involved became apparent, once again. All parties involved were inspected by different divisions within the same competent authority and a good mutual communication is essential. Figure 3 gives a peek view of all parties involved in a one-day production of the slaughterhouse. Risks related to contamination at this stage of the supply chain should not be underestimated and can lead to great health risk as well as economic consequences due to recall and destruction of incriminated batches, as shown in this case.

One third of cases involved in the current outbreak was 9 years or younger with the youngest case being two years old. Kivi et al.^21^ previously showed that younger age (0-9) is a particularly strong risk factor for *S*. Typhimurium infection (odds ratio 6.3; 95% confidence interval 1.7-23.6), possibly relating to a higher susceptibility of this age group. As the very young, elderly, pregnant and the immunocompromised are known to be at greatest risk of severe disease and death in relation to food- and waterborne infections^22^, these vulnerable groups should abstain from eating raw or undercooked meat products. Similar to already implemented mandatory provision of allergen information on food product labels^23^ – we recommend adding explicit warning labels to food products that could potentially be microbiologically unsafe for consumers, especially meat products that are eaten raw or undercooked. As was suggested previously^21^, adding such information, associated health risks would be transparent and would allow consumers to make well-informed decisions when consuming risk products.

In general, to ensure food safety, batches of products that are intended to be eaten without sufficient heat treatment, should be submitted to risk based HACCP procedures in order to verify compliance with regulations. If such standards are not met – or as a general part of the production process - pre-treating risk products prior to sale could be considered. Pre-treatment methods, such as high-pressure processing that applies pressure to food products and thereby inactivates microorganism without alteration of taste or texture of the food product, constitute promising methods for that purpose^24^.

In conclusion, this outbreak investigation highlighted the importance of close collaboration between regional Public Health Services, the NVWA, the RIVM and diagnostic laboratories. Through a combined microbiological, epidemiological and source tracing approach, raw and undercooked beef products could be identified as the source of the *S*. Typhimurium outbreak. This outbreak yet again emphasizes the importance of awareness among consumers of the risk of infection when consuming or handling raw meat products. To increase transparency for consumers, we recommend adding warning labels to risk food products.

## Competing Interests

The authors have declared that no competing interests exist.

## Data Availability Statement

Access to data are restricted to protect the confidentiality of individuals and premises involved in this outbreak. Researchers interested in accessing an anonymised minimal data set should write to the National Institute for Public Health and the Environment (details below), who will assess the request. Requests for data should be addressed to: Department Epidemiology and Surveillance of Gastroenteritis and Zoonoses (pb 75), Centre for Infectious Disease Control (CIb), National Institute for Public Health and the Environment (RIVM), Pbox 1, 3720 BA Bilthoven, the Netherlands (eelco.franz@rivm.nl).

## Corresponding Author

Ingrid Friesema: ingrid.friesema@rivm.nl
